# Association of immunologic findings of atheromatous plaques with subsequent cardiovascular events in patients with peripheral artery disease

**DOI:** 10.1038/s41598-023-50751-8

**Published:** 2024-01-03

**Authors:** Suh Min Kim, Soon Auck Hong, Jeong-Min Kim

**Affiliations:** 1 Department of Surgery, Chung-Ang University College of Medicine, Chung-Ang University Hospital, Seoul, South Korea; 2 Department of Pathology, Chung-Ang University College of Medicine, 102 Heukseok-Ro, Dongjak-Gu, Seoul, 06973 Republic of Korea; 3 Department of Neurology, Seoul National University Hospital, 101 Daehak-Ro, Jongno-Gu, Seoul, 03080 Republic of Korea

**Keywords:** Biomarkers, Cardiology

## Abstract

Patients with peripheral artery disease (PAD) have a higher risk of cardiovascular events. We examined the histology of atheromatous plaques in the femoral artery and investigated their association with subsequent cardiovascular events in patients with PAD. Patients who underwent femoral artery endarterectomy between March 2010 and January 2021 were included. We analyzed the expression of myeloperoxidase (MPO), citrullinated histone, and programmed cell death ligand 1 (PD-L1) in femoral artery plaques by immunohistochemistry. Data on the subsequent occurrence of major adverse cardiovascular events (MACEs), major adverse limb events (MALEs), and all-cause mortality were retrospectively collected. A total of 37 patients were included. The median age was 71 (range, 42–90) years, and 25 patients (67.6%) were male. During the median follow-up of 24 months, 10 patients experienced MACEs and 16 patients had MALEs. Patients with MACEs had a higher number of MPO-stained cells (*p* = 0.044) and lower PD-L1 staining intensity (*p* = 0.021) in atheromatous plaques compared with those of patients with a stable prognosis. When the patients were grouped according to the immunologic score based on the MPO-stained cell number and PD-L1 staining intensity, those with a higher score had a significantly higher cumulative risk of MACEs (*p* = 0.014). The immunologic profile of excised peripheral artery plaques may be associated with future cardiovascular events in patients with PAD.

## Introduction

The incidence of lower extremity peripheral artery disease (PAD) has increased, and over 200 million individuals worldwide are affected^1^. PAD has been recognized as an important cause of cardiovascular morbidity and mortality in addition to coronary artery disease and stroke^2,3^. The presence of atherosclerosis in more than two vascular beds is termed polyvascular disease^4^. The severity of atherosclerotic disease in one arterial bed is positively associated with disease in additional beds. PAD increases the risk of myocardial infarction and ischemic stroke^5^.

The role of the inflammatory process in the initiation and development of atherosclerosis is well known^6^. Proinflammatory activation of endothelial cells, capture of circulating monocytes and T cells, and proinflammatory cytokines secretion by plaque macrophages are process involved in disease initiation^7^. Various inflammatory molecules have been investigated as biomarkers and therapeutic targets. Neutrophils are the most abundant leukocytes and play a crucial role in the innate immune response^8^. Activated neutrophils release a web-like structure composed of DNA and citrullinated histones, known as neutrophil extracellular traps (NETs)^9^. The role of NETs has been investigated in coronary artery disease and acute ischemic stroke^10,11^. The programmed cell death protein-1/programmed death-ligand 1 (PD-1/PD-L1) pathway is important for immune system inhibition^12^. Although, the association between PD-L1 expression and acute coronary syndrome has been reported, little is known about the role of PD-L1 in lower limb arterial disease^13^.

A histologic analysis of inflammatory markers in atheromatous plaques and thrombi can provide insights into the mechanism of PAD; however, only a few studies have conducted a histologic analysis of atheromatous plaques, mostly in carotid or coronary artery disease^14–16^. The immunologic phenotype of thrombi from stroke patients, such as increased NETs and decreased PD-L1 expression, and the absence of high mobility group box 1 was associated with further vascular event after index stroke^17^. We analyzed the expression of neutrophils, NETs, and PD-L1 in femoral artery plaques after endarterectomy and investigated their association with subsequent cardiovascular events.

## Methods

### Patient inclusion and data collection

Consecutive patients who underwent femoral artery endarterectomy between March 2010 and January 2021 and whose atheromatous plaque specimen was available were retrospectively included. The indications for revascularization were severe claudication persistent even after best medical therapy and exercise therapy or chronic limb threatening ischemia. Patients who underwent additional procedures, such as endovascular stenting and arterial bypass surgery, were included.

Data on baseline demographics, comorbidities, and current medications including statins, antiplatelet agents, and anticoagulants were collected retrospectively. The preoperative ankle-brachial index was measured. All patients underwent computed tomography (CT) angiography before the surgical procedure. Detailed information on the surgical procedure, operative time, and additional surgical or endovascular procedure was collected. Endpoints included major adverse cardiovascular events (MACEs), major adverse limb events (MALEs), and all-cause mortality. MACEs were defined as myocardial infarction, stroke, or death from a cardiovascular cause. MALEs were defined as restenosis of the index lesion (> 50% stenosis in CT angiography or duplex ultrasonography), index-limb revascularization, or ipsilateral limb amputation for a vascular cause.

This study was approved by the Chung-Ang University Hospital Institutional Review Board (IRB no. 2070-001-422, 2202-003-495) and performed in accordance with the Declaration of Helsinki. Written informed consent was obtained from the participants.

### Histological analysis

The excised plaques specimens were fixed with 10% neutral-buffered formalin and embedded in paraffin. The formalin-fixed, paraffin-embedded blocks were dissected into 4-μm sections. Hematoxylin and eosin (H&E) staining was performed, using Leica ST5010 Autostainer XL (Leica, Wetzlar, Germany). The average number of white blood cells (WBCs) including neutrophils, lymphocytes, and macrophages in 3 high-power fields (× 400), which showed the highest number of cells, was calculated by a pathologist (Supplementary Fig. 1). The presence of luminal thrombi and calcification was also analyzed.

### Immunohistochemical staining and immunologic scores

Immunohistochemical staining was performed for the expression of myeloperoxidase (MPO), citrullinated histone (H3Cit), and PD-L1 using a Ventana BenchMark autostainer (Ventana Medical Systems, Tucson, AZ, USA). In brief, formalin-fixed, paraffin-embedded sections were deparaffinized and antigen retrieval was performed using MC1 solution (Ventana Medical Systems, Tucson, AZ, USA). The sections were incubated with the following primary antibodies; MPO (pre-dilution, clone SP72; Roche, Tucson, AZ, USA), H3Cit (1:100, clone ab5103; Abcam, Cambridge, MA, USA), and PD-L1 (pre-dilution, clone SP263, Roche, Tucson, AZ, USA). The ultraView polymer detection kit (Ventana Medical Systems, Tucson, AZ, USA) was used for visualization. Nonimmune normal IgG was used to replace primary antibodies as a negative control, and no staining occurred.

The average number of stained cells for MPO and H3Cit in 3 high-power fields (× 400) was calculated by a pathologist (Supplementary Fig. 2). The scores for MPO were as follows: 0 (0–99 positive cells); 1 (100–299 positive cells); and 2 (> 300 positive cells). PD-L1 expression was evaluated based on the intensity and the area of the staining (Supplementary Fig. 3). The scores for PD-L1 were defined as follows: 0 (intense reaction), 1 (moderate reaction), 2 (mild reaction), and 3 (no color reaction). The area of PD-L1 expression was evaluated as the percentage of the stained area of the entire atheroma specimen. The immunologic score of MPO and PD-L1 was defined as the sum of each score (Supplementary Table 1).

### Statistical analysis

Continuous data are summarized as medians with the range and compared by the Mann–Whitney test. Categorical data are summarized as proportions and percentages and compared by the chi-square test. Receiver operating characteristic (ROC) analysis was performed to analyze the association of histologic markers with MACEs and to determine an appropriate cutoff value for further dichotomized analysis. The Kaplan–Meier method was used to estimate freedom from MACEs. Cox regression analysis was performed to determine independent variables associated with MACE- free survival and all-cause mortality. Significant variables from bivariable analyses were considered for inclusion in the final multivariable model. A *p* value of < 0.05 was considered statistically significant. All statistical analyses were performed using IBM SPSS ver. 26.0 (IBM Co., Armonk, NY, USA).

## Results

### Baseline and clinical characteristics of patients

A total of 37 patients were included in the study. The median age was 71 (range, 42–90) years and 25 (67.6%) patients were male. The majority of the patients (n = 28, 75.7%) underwent endarterectomy only without additional procedures. During the median follow-up of 24 months (range 1–132), 10 patients experienced MACEs, 16 patients had MALEs and 6 patients had both. Among the 10 patients with MACE, 6 were diagnosed with stroke and 4 were treated as coronary artery disease. The patients were grouped according to the occurrence of MACEs after index femoral endarterectomy. Patients with MACEs were more likely to have a history of coronary artery disease (60.0% vs. 14.8%, *p* = 0.012) and stroke (50.0% vs. 14.8%, *p* = 0.041). There was no difference in the clinical severity, concomitant procedures with endarterectomy, operative time, and hospital stay (Table 1). During follow-up, 12 patients died; 3 patients due to infection, 2 due to malignancy and 1 due to heart failure and the cause of death was not identifiable in 6 patients. Among the 12 patients, 3 underwent adjunctive procedures, such as balloon angioplasty or stent insertion, simultaneously with endarterectomy. Two patients underwent additional endovascular and surgical intervention during follow-up; one underwent thrombectomy of contralateral limb 2 days after the initial operation and the other underwent iliac artery stenting 5 year after the endarterectomy.

**Table 1:** Baseline characteristics of patients.

Variables	Major adverse cardiovascular events (n = 10)	No major adverse cardiovascular events (n = 27)	*P*
Age, median with range (yr.)	69 (51–90)	71 (42–87)	0.625
Sex, male (%)	5 (50.0%)	20 (74.1%)	0.240
Comorbidities, n (%)			
Hypertension	8 (80.0%)	19 (70.4%)	0.694
Diabetes mellitus	4 (40.0%)	8 (29.6%)	0.696
Coronary artery disease	6 (60.0%)	4 (14.8%)	0.012
Stroke	5 (50.0%)	4 (14.8%)	0.041
Atrial fibrillation	5 (50.0%)	6 (22.2%)	0.125
Chronic kidney disease	1 (10.0%)	3 (11.1%)	0.528
Smoking, n (%)			
Current	2 (20.0%)	7 (25.9%)	0.064
Ex-smoker	1 (10.0%)	12 (44.4%)	
Never	7 (70.0%)	8 (29.6%)	
Anticoagulation at admission	2 (20.0%)	4 (14.8%)	0.663
Antiplatelet agent at admission	5 (50%)	13 (48.1%)	0.666
Statin at admission	3 (30.0%)	7 (25.9%)	0.999
Clinical manifestation			
Intermittent claudication	2 (20.0%)	13 (48.1%)	0.208
CLTI	8 (80%)	14 (51.9%)	
Ankle-brachial index, index limb	0.46 (0–0.49)	0.54 (0–0.72)	0.082
Adjunctive procedure			
Balloon angioplasty	0	2 (7.4%)	0.839
Stent insertion	1 (10.0%)	3 (11.1%)	
Bypass surgery	1 (10.0%)	2 (7.4%)	
Operation time, (min.)	95 (45–470)	180 (70–575)	0.180
Hospital stay, (days)	15 (3–89)	9 (4–117)	0.749
Antithrombotics after operation			
Anticoagulant	3 (30.0%)	5 (18.5%)	0.208
Single antiplatelet agent	3 (30.0%)	2 (7.4%)	
Dual antiplatelet agents	3 (30.0%)	16 (59.3%)	
Anticoagulant and antiplatelet agent	1 (10.0%)	4 (14.8%)	
Laboratory findings			
Total cholesterol (mg/dL)	135 (88–278)	169.5 (94–294)	0.288
hs-CRP (mg/dL)	0.8 (0.3–4.6)	1.3 (0.13–52.1)	0.556
Neutrophil (μL)	3488 (2200–11,719)	4300 (249–12,050)	0.494

*DOAC* direct oral anticoagulants, *CLTI* chronic limb threatening ischemia.

Histologic evaluation with H&E staining showed that the patients with MACEs or MALEs had a similar number of WBCs in atheromatous plaques compared with the number of those with a stable prognosis (median (range), 346 (6–617) vs. 117 (0–449), *p* = 0.111 and 82 (0–617) vs. 204 (1–426), *p* = 0.506, respectively). In addition, there was no significant difference in the expression of WBCs in the femoral artery plaques of patients grouped according to the all-cause mortality. The presence of calcification or luminal thrombi was not significantly different between groups with and without MACEs (*p* = 0.461 and *p* = 0.274, respectively) or MALEs (*p* = 0.519 and *p* = 0.739, respectively).

### Expression of MPO, NETs, and PD-L1

**Figure 1:**
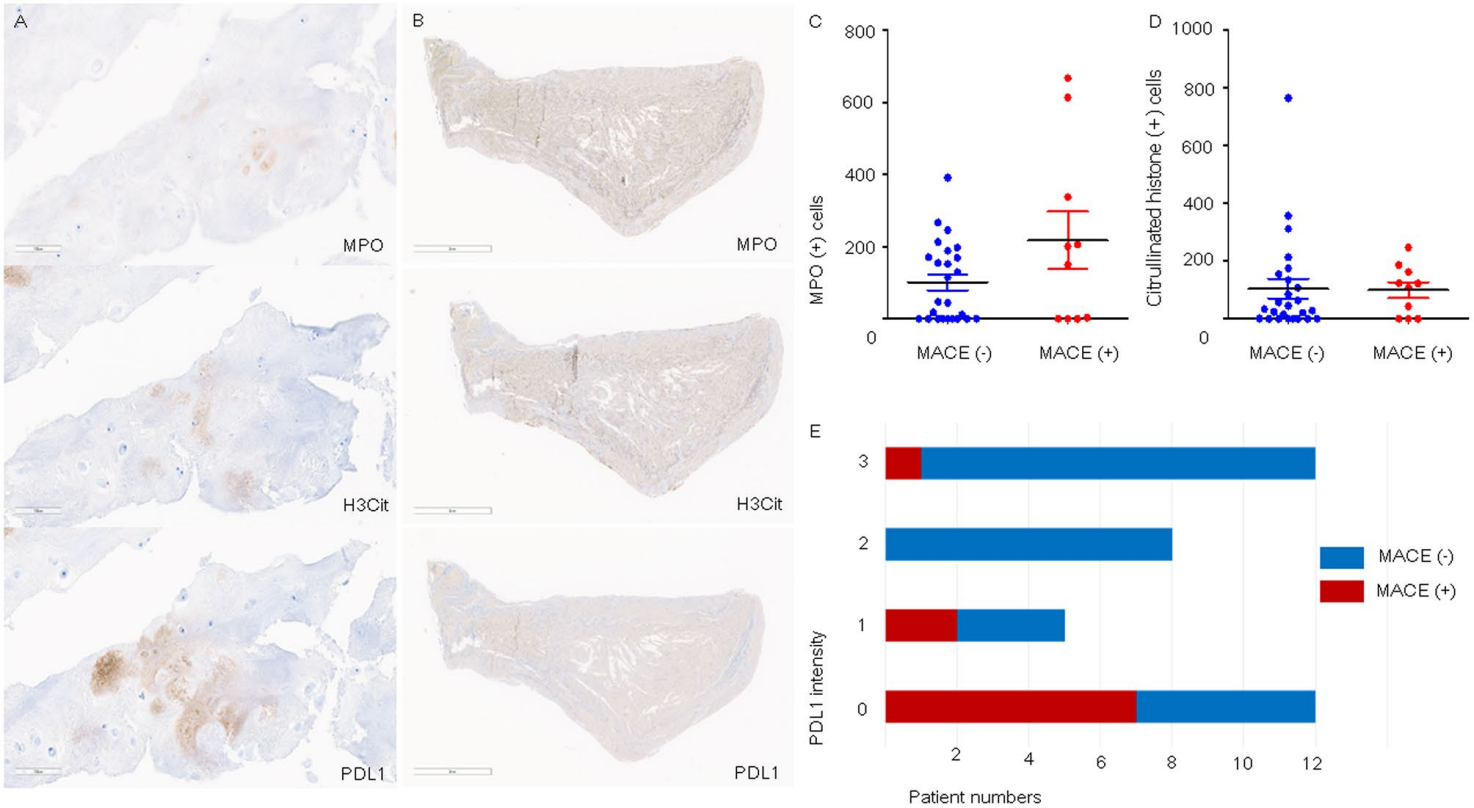
Representative histologic images of an atheromatous plaques and comparison of immunologic markers. (**A**) An atheroma from a patient with stable prognosis showed a reduced number of myeloperoxidase (MPO)-positive cells and enhanced programmed cell death ligand (PD-L1) expression intensity compared with (**B**) the MPO-stained cells and PD-L1 expression of an atheroma from a patient who experienced stroke after femoral endarterectomy. (**C**) A comparison of immunologic markers between patients with and without major adverse cardiovascular events (MACEs) showed that patients with vascular events had a significantly higher number of MPO-positive cells. (**D**) The number of citrullinated histone (H3Cit)-positive cells was not different between the two groups. (**E**) The PD-L1 expression intensity of the atheromatous plaques was associated with the occurrence of MACEs after femoral endarterectomy.

A representative histologic specimen without further vascular events had a reduced number of MPO-positive cells, but more intense PD-L1 expression (Fig. 1A) compared with those of a patient who suffered from stroke after femoral endarterectomy (Fig. 1B). Patients with MACEs had a higher number of MPO-positive cells (median (range), 205 (6–718) vs. 89 (0–377), *p* = 0.044, Fig. 1C) and lower PD-L1 intensity (median (range), 1 (0–3) vs. 3 (0–3), *p* = 0.021, Fig. 1E) in atheromatous plaques compared with those of patients with a stable prognosis. The number of H3Cit-positive cells (median (range), 122 (0–247) vs. 108 (0–765), *p* = 0.370, Fig. 1D) and the PD-L1 expression area (median (range), 10 (0–35) vs. 5 (0–40), *p* = 0.072) were not significantly different between the two groups. There was no difference in the expression of MPO, H3Cit, and PD-L1 between patients with and without MALEs (Supplementary Table 2). In addition, the three immunologic markers were not associated with all-cause mortality (Supplementary Table 2).

### Factors associated with MACEs

**Figure 2:**
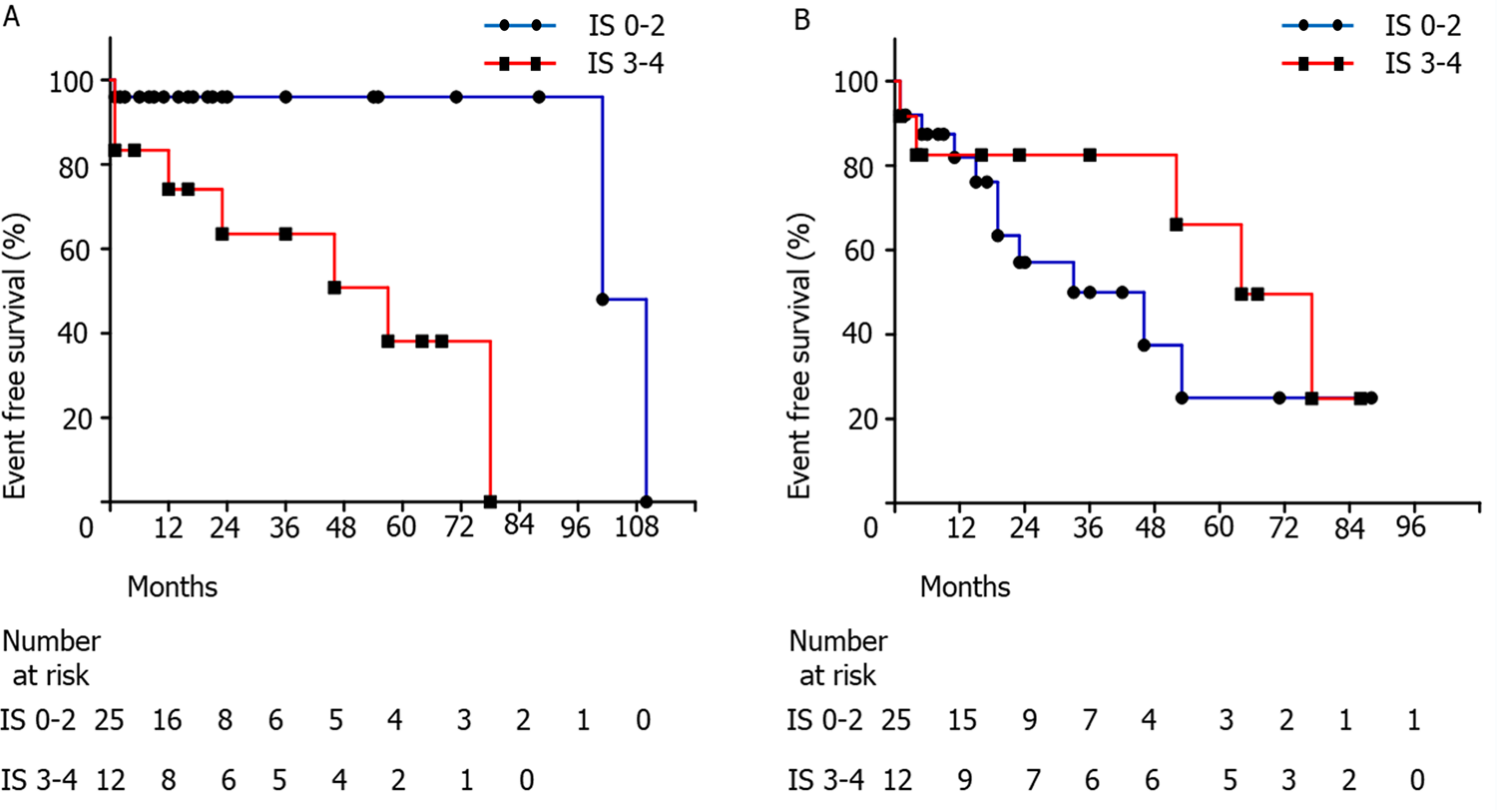
Survival curve analysis according to the immunologic score. (**A**) The patient group with higher immunologic score (red line) had significantly shorter major adverse cardiovascular events (MACEs)-free survival than that of the patient group with lower immunologic score (blue line, log-rank test, *p* = 0.014, (**A**)). (**B**) The occurrence of major adverse limb events (MALEs) was not significantly different between the two groups (log-rank test, *p* = 0.298). *IS* immunologic score.

Patients with MACEs had a significantly higher immunologic score compared with that of patients without MACEs (median (range), 3 (1–5) vs. 1 (0–5), p = 0.006). Patients were classified into two groups according to the immunologic score; 0–2, lower risk group (n = 22) and 3–5, higher risk group (n = 15) after deriving the appropriate cutoff value of the immunologic score from ROC analysis (Supplementary Fig. 4). The Kaplan-Meier estimate of MACE-free survival was significantly lower in the higher risk group (p = 0.014) (Fig. 2A). A cox proportional hazard model including previous stroke and immunologic score revealed that stroke was significantly associated with MACEs (hazard ratio, 5.11; 95% confidence interval 1.21–21.63; p=0.027), and a higher immunologic score tended to be associated with the cumulative risk of MACEs (hazard ratio, 4.78; 95% confidence interval, 1.97–23.44; p = 0.054) (Table 2). The immunologic score was not associated with the occurrence of MALEs and all-cause mortality (Fig. 2B, Supplementary Table 2).

**Table 2:** Factors associated with major adverse cardiovascular events (MACEs).

	Bivariable			Multivariable		
	HR	95% CI	*P*	HR	95% CI	*P*
Age	1.02	0.97–1.08	0.456			
Hypertension	1.39	0.29–6.72	0.679			
Diabetes Mellitus	1.18	0.29–4.77	0.818			
Coronary artery disease	2.93	0.75–11.41	0.121			
Stroke	6.17	1.46–26.07	0.013	5.11	1.21–21.63	0.027
Atrial fibrillation	3.55	0.94–13.42	0.062			
Preoperative ABI of index limb	0.01	0.00–2.14	0.095			
Smoking	0.52	0.10–2.58	0.420			
hs-CRP	0.90	0.58–1.40	0.631			
Immunologic scores for MPO and PDL-1 ≥ 2	5.58	1.16–26.93	0.032	4.78	1.97–23.44	0.054

*HR* hazard ratio, *CI* confidence interval, *ABI* ankle brachial index, *CRP* c-reactive protein, *MPO* myeloperoxidase, *PDL-1* programmed death ligand-1.

## Discussion

This study demonstrated the association of the immunologic findings of femoral artery plaques with future cardiovascular events. The major finding of the study was that the femoral artery atheromatous plaques of patients with MACEs were associated with a higher number of MPO-expressing cells and lower PD-L1 intensity compared with those of patients without MACEs. The score based on the MPO-stained cell number and PD-L1 staining intensity was associated with future vascular events after femoral artery endarterectomy.

Neutrophils are known to play a key role in plaque development, erosion, and rupture and are associated with plaque instability in atherosclerosis^18–20^. MPO is one of the main enzymes released following neutrophil activation^21^. In particular, MPO converts low-density lipoprotein into an atherogenic form, generates various reactive oxidants, and reduces nitric oxide availability, which contributes to endothelial dysfunction^22^. Some studies have demonstrated the association of serum MPO level with PAD or cardiovascular events; however, data from the direct analysis of excised atheromatous plaques are limited^22,23^. Based on the findings of this study, an increased number of MPO-stained cells within femoral artery plaques may be a candidate predictor for systemic cardiovascular risks.

NET formation has been identified as a novel mechanism in addition to classic neutrophil functions, such as degranulation, cytokine release, and oxidative burst^24^. Activated neutrophils recruited to plaques can exacerbate atheroprogression through NET formation. The association between NETs and coronary or carotid artery disease has been investigated. Mangold et al. found that the coronary NET burden in acute coronary syndrome was a predictor of myocardial infarct size^25^. Navotny et al. showed that the amount of NETs was associated with the outcomes of patients with ischemic stroke and myocardial infarction^10^. Our group found that higher NET burden in thrombi from stroke patients was associated with future vascular event^17^. However, the association between the burden of NETs and vascular event was not significant in this study, probably due to the heterogeneous nature of excised atheroma specimen and the small number of included patients. Further studies with a larger cohort are warranted to establish the clinical significance of NETs in PAD.

Signaling through the coinhibitory PD-1/ PD-L1 pathway regulates T cell responses and plays a key role in maintaining the immune tolerance and immune evasion of cancers and pathogens^26^. In atherosclerosis, PD-L1 has been shown to decrease the production of proinflammatory cytokines and attenuate the activation of T cells, suggesting an atheroprotective effect^27^. Reducing the excessive immune response during atherosclerosis progression by inducing PD-1/PD-L1 signaling may have a therapeutic effect^28^. Recently, a higher rate of cardiovascular events after using immune checkpoint inhibitors was reported, which indirectly demonstrated the role of the PD-1/PD-L1 pathway in atherosclerosis^29^. Several studies have investigated the association between the blood level of soluble PD-L1 and acute coronary syndrome^13,30^. However, studies on the tissue expression of PD-L1 in atherosclerotic disease are limited. The level of soluble PD-L1 in peripheral blood may be affected by various factors, such as infection or autoimmunity; thus, the tissue expression of PD-L1 could be a more effective prognostic marker.

Atherosclerosis is a systemic disease that may involve multiple vascular territories. In PAD patients, myocardial infarction or stroke is the leading cause of mortality; thus, predicting their risk is important^31^. The immunologic score based on MPO-stained cells and PD-L1 intensity could be used to identify patients who require further diagnostic evaluation for coronary or carotid artery disease and select patients for more aggressive antithrombotic therapy or surveillance after peripheral artery revascularization. Neutrophils are important cellular components of the innate immunity, and PD-L1 is associated with T cell activation and the adaptive immune system. Various immune cells play a vital role in atherosclerosis plaques, and both adaptive and innate immune systems are involved in the pathogenesis of atherosclerosis^32^. Moreover, there are crucial proteins to mediate the initiation and progression of inflammatory process in atherosclerosis. Proprotein convertase subtilisin/kexin type 9 (PCSK9) is a key driver of the atherosclerosis process by increasing plasma low density lipoprotein and inducing endothelial dysfunction. PCSK9 inhibitor has emerged as novel therapy to treat immune checkpoint inhibitors-related atherosclerotic cardiovascular disease^33^. It is worth investigating the association between the expression of the proteins related to immune response in atheromatous plaque and future vascular events.

Our study had several limitations. First, this was a retrospective study with a small number of patients in a single center, which may have limited statistical power. Further validation of the predictive value of MPO and PD-L1 for future cardiovascular events with a larger prospective cohort is warranted. Second, we proposed specific immunologic markers based on the literature review and pathophysiologic insights of atherosclerosis, however, more useful biomarker candidates could be derived from unbiased proteomic analysis. Further mechanistic studies will be needed to clarify the role of PD-L1 in atherosclerosis.

In conclusion, the immunologic profile of excised peripheral artery plaques was associated with future cardiovascular events in patients with PAD. Specifically, the score based on the MPO-stained cell number and PD-L1 staining intensity in femoral artery atheromatous plaques was associated with subsequent cardiovascular events after femoral artery endarterectomy. The higher number of infiltrated neutrophils and weak immune checkpoint signal intensity in peripheral atheromatous plaques may be markers for cardiovascular events. Further studies with a larger cohort are needed to validate the candidate predictive markers.

### Supplementary Information

Supplementary Figures.

Supplementary Tables.

## Data Availability

The datasets generated and/or analyzed during this study are available from the corresponding author upon reasonable request.
