# Supplementary material for: Association of immunologic findings of atheromatous plaques with subsequent cardiovascular events in patients with peripheral artery disease
Source: Sci Rep. 2024 Jan 3;14:469. doi: 10.1038/s41598-023-50751-8 (PMC10764821; doi:10.1038/s41598-023-50751-8)
Supplement: Supplementary file 1 — Supplementary Figures. [file 41598_2023_50751_MOESM1_ESM.docx]

**Supplementary Figure 1**. Representative image of atheromatous plaques showing diverse histologic phenotypes. The number of inflammatory cells was difference according to the specimens and observed field. (H&E stain) (A-D, magnification ×400)

**Supplementary Figure 2**. Representative images of immunohistochemical staining for myeloperoxidase (MPO): (A) no stained cells, (B) score = 0; 0–99 positive cells, (C) score = 1; 100–199 positive cells, and (D) score = 2; > 200 positive cells (×400).

S**upplementary Figure 3.** Representative images of immunohistochemical staining for programmed cell death ligand 1 (PD-L1): (A,B) no color reaction, (C,D) weak expression, (E,F) intermediate expression, and (G,H) intense reaction (magnification ×100 and ×400, respectively)

**Supplementary Figure 4**. Receiver operating characteristic (ROC) analysis and definition of an appropriate cutoff value for the immunologic scores for predicting major adverse cardiovascular events (MACEs).

**Supplementary Figure 1**. Representative image of atheromatous plaques showing diverse histologic phenotypes. The number of inflammatory cells was difference according to the specimens and observed field. (H&E stain) (A-D, magnification ×400)


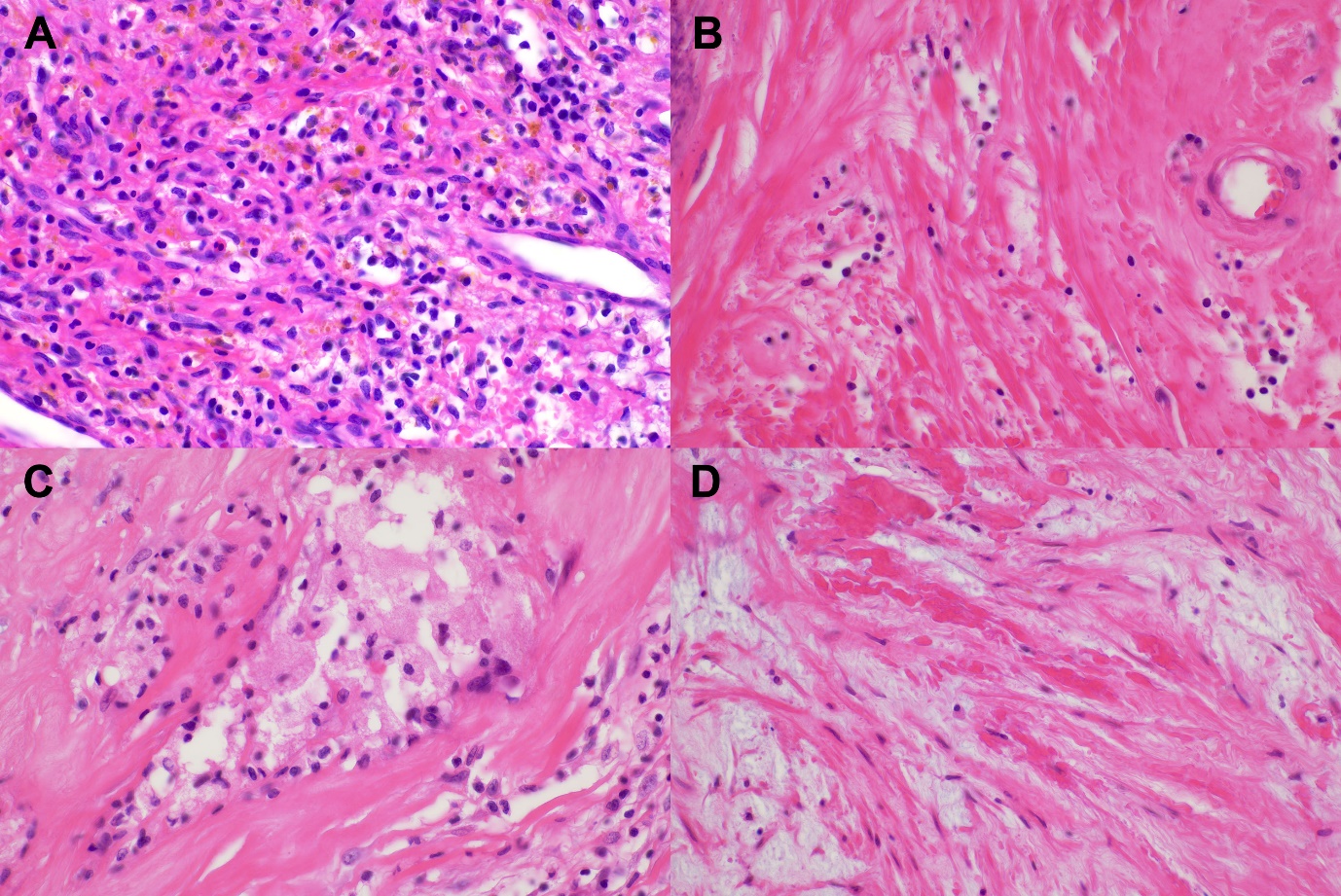


**Supplementary Figure 2**. Representative images of immunohistochemical staining for myeloperoxidase (MPO): (A) no stained cells, (B) score = 0; 0–99 positive cells, (C) score = 1; 100–199 positive cells, and (D) score = 2; > 200 positive cells (×400).

**
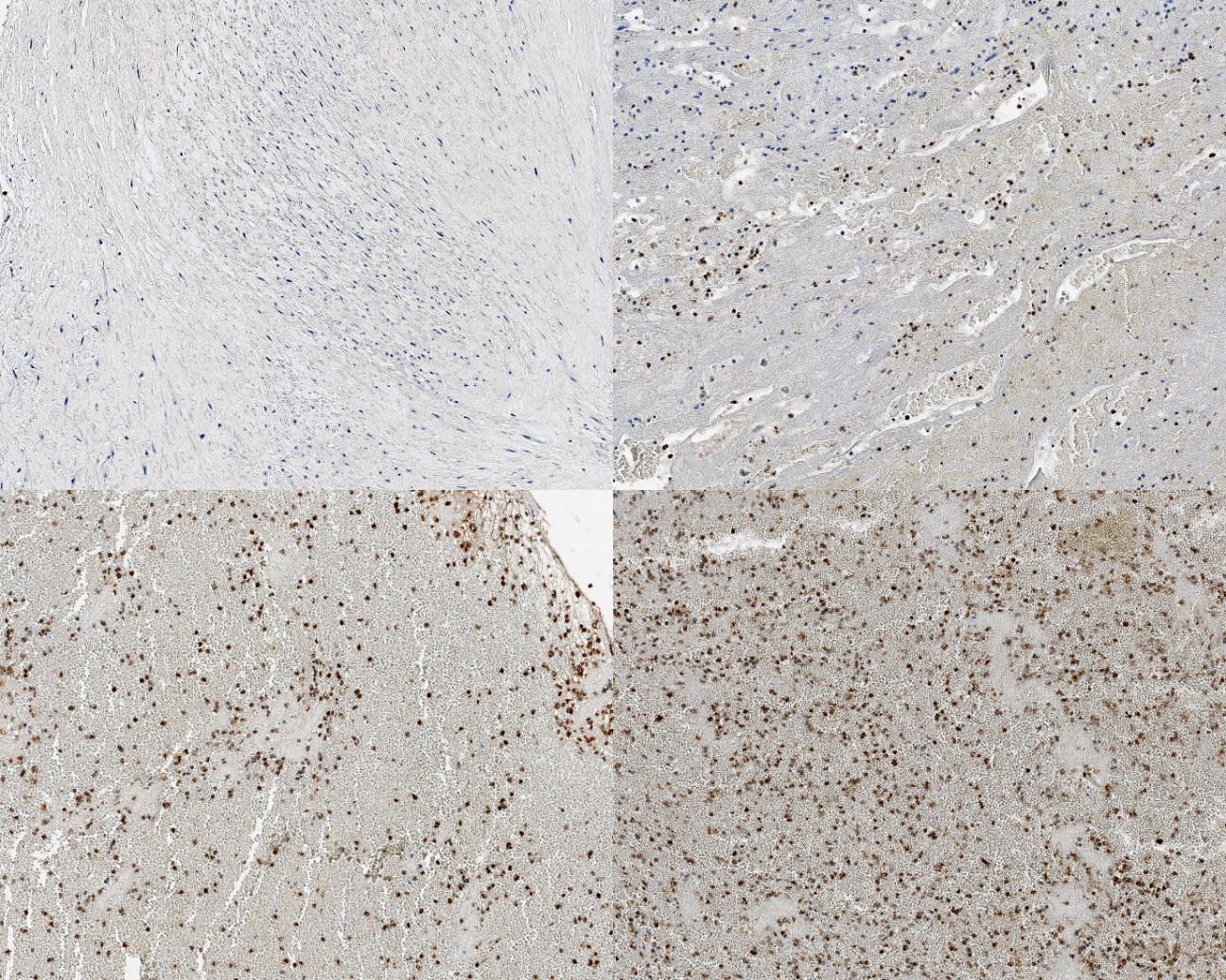
**

**B**

**C**

**D**

**A**

**Supplementary Figure 3.** Representative images of immunohistochemical staining for programmed cell death ligand 1 (PD-L1): (A,B) no color reaction, (C,D) weak expression, (E,F) intermediate expression, and (G,H) intense reaction (magnification ×100 and ×400, respectively)
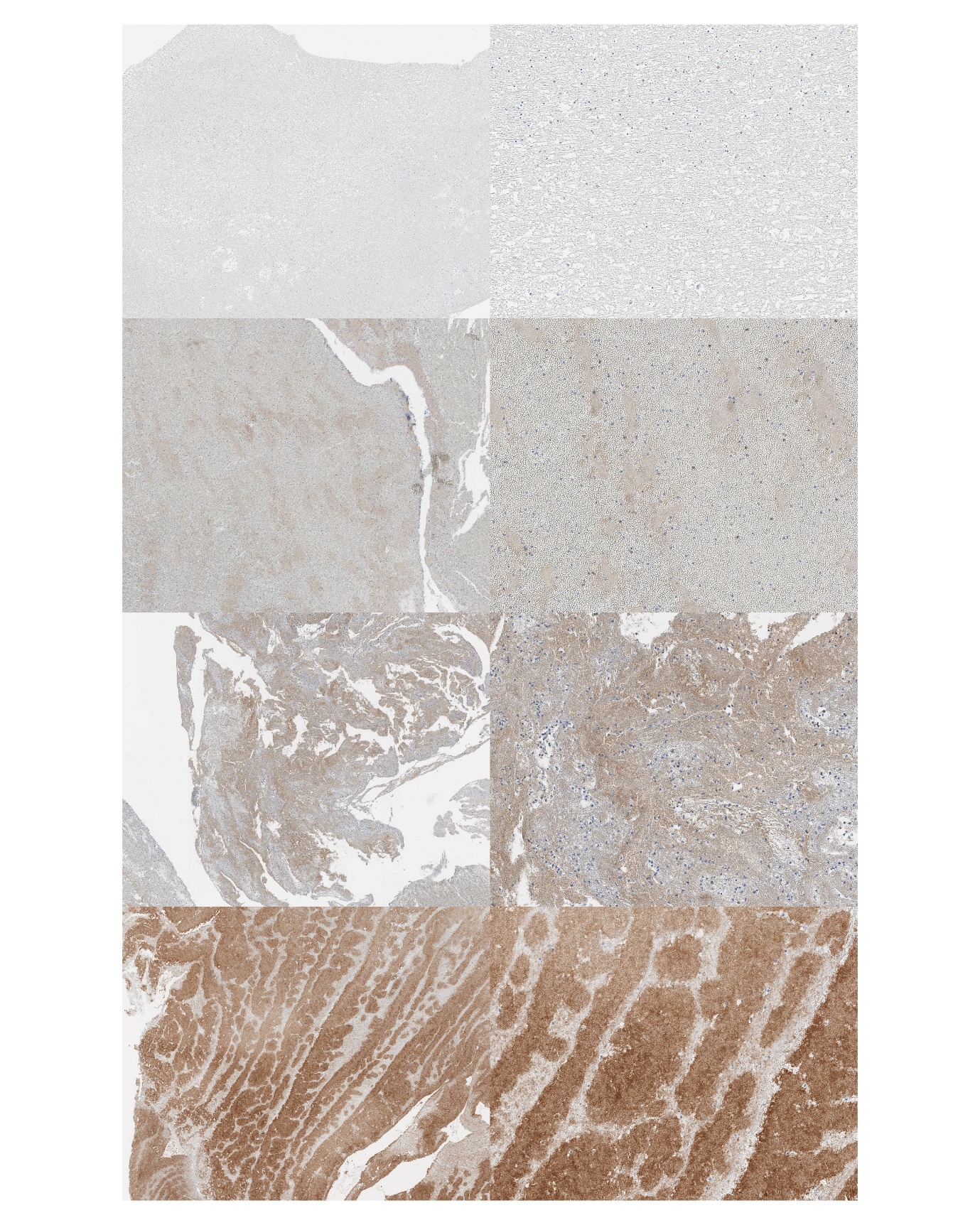


**H**

**G**

**F**

**EE**

**D**

**C**

**B**

**A**

**Supplementary Figure 4**. Receiver operating characteristic (ROC) analysis and definition of an appropriate cutoff value for the immunologic scores for predicting major adverse cardiovascular events (MACEs).


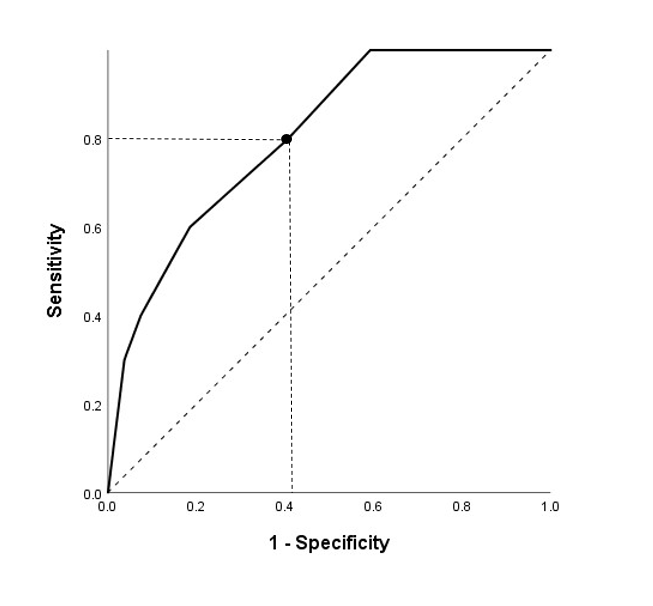


AUC 0.804

***P*** = 0.005
