# Supplementary material for: Association of immunologic findings of atheromatous plaques with subsequent cardiovascular events in patients with peripheral artery disease
Source: Sci Rep. 2024 Jan 3;14:469. doi: 10.1038/s41598-023-50751-8 (PMC10764821; doi:10.1038/s41598-023-50751-8)
Supplement: Supplementary file 2 — Supplementary Tables. [file 41598_2023_50751_MOESM2_ESM.docx]

Supplementary Table 1. Immunologic score based on MPO and PD-L1.

Supplementary Table 2. Association of the histopathologic findings of femoral artery plaques with major adverse limb events (MALEs) and all-cause mortality

**Supplementary Table 1 Immunologic scores based on MPO and PD-L1.**

| **Scores for MPO** | **Scores for PDL-1 intensity** |
| --- | --- |
| 0 = 0-99 positive cells | 0 = positive, intense expression; intense reaction |
| 1 = 100-299 positive cells | 1 = positive, intermediate expression ; moderate reaction |
| 2 = >300 positive cells | 2 = positive, weak expression ; mild reaction |
|  | 3 = negative, no color reaction |

**Supplementary Table 2. Association of the histopathologic findings of femoral artery plaques with major adverse limb events and all-cause mortality**

|  | **Major adverse limb events**  **(n=16)** | **No major adverse limb events (n=21)** | ***P*** |
| --- | --- | --- | --- |
| MPO | 78 (0-590) | 168 (0-416) | 0.138 |
| CitH3 | 35 (0-765) | 84 (0-356) | 0.215 |
| PDL-1 | 3 (0-3) | 2 (0-3) | 0.144 |
| Immunologic scores | 1 (0-5) | 1 (0-5) | 0.461 |
|  | **All-cause mortality (n=12)** | **Control (n= 25)** | ***P*** |
| MPO | 268 (5-590) | 67 (0-416) | 0.323 |
| CitH3 | 108 (0-356) | 44 (0-765) | 0.244 |
| PDL-1 | 2 (0-3) | 2 (0-3) | 0.467 |
| Immunologic scores | 2 (0-5) | 1 (0-5) | 0.414 |

§MPO, myeloperoxidase; CitH3, citrulinated histone H3; PDL-1, programmed death ligand-1
